# Pancreatic Volumetric Assessment as a Predictor of New-Onset Diabetes Following Distal Pancreatectomy

**DOI:** 10.1007/s11605-012-2039-7

**Published:** 2012-09-28

**Authors:** Sachiyo Shirakawa, Ippei Matsumoto, Hirochika Toyama, Makoto Shinzeki, Tetsuo Ajiki, Takumi Fukumoto, Yonson Ku

**Affiliations:** Division of Hepato-Biliary-Pancreatic Surgery, Department of Surgery, Kobe University Graduate School of Medicine, 7-5-2 Kusunoki-cho, Chuo-ku, Kobe, Hyogo 650-0017 Japan

**Keywords:** Pancreas, Volumetry, Pancreatic diabetes

## Abstract

**Introduction:**

Pancreatogenic diabetes after pancreatectomy is of growing importance due to the increasing life expectancy of pancreatectomized patients. Although reduction of pancreatic volume is thought to affect glucose metabolism, a consistent relationship has yet to be determined. This study aimed to investigate functional consequences of distal pancreatectomy (DP) in preoperatively non-diabetic patients.

**Methods:**

This study included 61 non-diabetic patients who underwent DP. Clinical data were obtained, and the percent resected volume (PRV) of each pancreas was determined via multi-detector row computed tomography volumetry.

**Results:**

During the follow-up period (median 26 months), 22 patients (36 %) developed new-onset diabetes within a median onset time of 8 months (range 0.5–42 months) postoperatively. The remaining 39 patients also showed impaired glucose metabolism. Multivariate analysis identified preoperative hemoglobin A1c ≥ 5.7 % (odds ratio 15.6, *p* = 0.001) and PRV > 44 % (odds ratio 11.3, *p* = 0.004) as independent risk factors for new-onset diabetes.

**Conclusions:**

Key determinants of postoperative glycemic control include preoperative functional reserve of the endocrine pancreas and the volume reduction of pancreatic parenchyma. Our findings enable reliable preoperative evaluation of the risk of postoperative diabetes and appropriate postoperative surveillance, which is helpful for early intervention in high risk patients.

## Introduction

Pancreatogenic diabetes, classified as type 3c by the American Diabetes Association,^1^ is associated with diseases of the exocrine pancreas including pancreatitis, benign and malignant neoplasm, cystic fibrosis, hemochromatosis, fibrocalculous pancreatopathy, and trauma and pancreatectomy. Among the 8–9 % of the general diabetes population with type 3c diabetes in Western countries, 2–3 % are those who underwent pancreatectomy.^2^,^3^ Pancreatectomized patients are at high risk for type 3c diabetes, as well as type 2, because surgery inevitably results in a deficit in the exocrine and endocrine pancreas, and also can promote the progression of underlying disease. Due to improved diagnostic modalities and a more refined understanding of pancreatic neoplasm pathogenesis, pancreatectomies for benign or low-grade malignant tumors are more frequent, and the life expectancy of patients undergoing pancreatectomy has increased in recent years. As the frequency of pancreatectomy and length of life expectancy increase, so does the importance of the risk of pancreatogenic diabetes associated with pancreatic surgery.

Distal pancreatectomy (DP) is the standard procedure used for removal of lesions in the body and tail of the pancreas. Long-term disturbances in glucose metabolism are a major concern after DP because previous studies have found that postoperative diabetes develops in from 4.8 to 38 % of patients after DP.^4^–^8^ Physiological factors reported to correlate with postoperative pancreatic endocrine function include preoperative fasting plasma glucose (FPG), body mass index (BMI), and postoperative complications.^9^–^11^ Limitations in these studies, such as unspecified preoperative diabetic status of the patients and inconsistent standards used for the diagnosis of postoperative diabetes, make it difficult to reliably identify risk factors for postoperative diabetes.

Although the mass of pancreatic beta cells has been identified as an important determinant of plasma glucose levels in rodents, dogs, monkeys, and humans,^12^–^15^ to our knowledge, very few studies have directly investigated the volume reduction of human pancreatic parenchyma as a risk factor for diabetes, and no previous study systematically quantified resection volumes in a population of patients. To study potential risk factors for new-onset diabetes in preoperatively non-diabetic patients, we sought to reliably quantify the volume reduction of human pancreatic parenchyma and to determine its longitudinal metabolic consequences following DP using multi-detector row computed tomography (MDCT) imaging volumetry.

## Methods

### Patients

A series of 98 consecutive patients who underwent DP at our institution between January 2005 and December 2011 was originally chosen from our prospectively maintained clinical database for this retrospective study. Data from 37 (38 %) of these candidates were excluded due to preoperative diabetes, as defined either by the WHO criteria of FPG ≥ 126 mg/dl detected on two or more separate days, or this abnormal FPG level detected once and plasma glucose ≥ 200 mg/dl measured 2 h after a 75-g glucose drink, or based on their treatment with oral anti-diabetic agents or insulin. The final study population consisted of 61 non-diabetic patients who had undergone DP.

Clinical data on pre- and postoperative patient status were obtained from existing medical records. Family histories of type 2 diabetes in first-degree relatives were also obtained. The preoperative data used for this study had been recorded within 14 days prior to surgery. Nutritional status and pancreatic endocrine functions were assessed based on measurements of body weight, serum albumin, FPG, and serum hemoglobin A1c (HbA1c). HbA1c values represent the National Glycohemoglobin Standardization Program (NGSP) equivalent values (in percent) and in all cases were converted from previous Japan Diabetes Society standard substance and measurement methods (JDS HbA1c, in percent) using the following formula: NGSP HbA1c (%) = JDS HbA1c (%) + 0.4 %. The percent resected volume (PRV) of pancreatic parenchyma, excluding tumor volume, was determined from abdominal MDCT measurements. Patient data were collected until the time of diagnosis of new-onset diabetes or tumor recurrence. All 61 patients were followed up for at least 3 months.

For evaluating postoperative course, we defined and graded postoperative pancreatic fistula (POPF) using the classification methods of the International Study Group of Pancreatic Fistula,^16^ with POPF grade B or C defined as clinically important pancreatic fistula. Postoperative complications were designated as level I to V based on the Clavien classification.^17^

### Determination of PRV of the Pancreatic Parenchyma

PRVs were determined retrospectively, using preoperative MDCT images in all patients. Continuous 0.8-mm 64-row MDCT images were acquired following administration of intravenous contrast material prior to surgery. MDCT data were transferred to a computer workstation (Aquarius; Elk, Osaka, Japan) for measurement of pancreas volume. To delineate the actual pancreatic resection lines, we compared preoperative CT with postoperative CT.

**Fig. 1:**
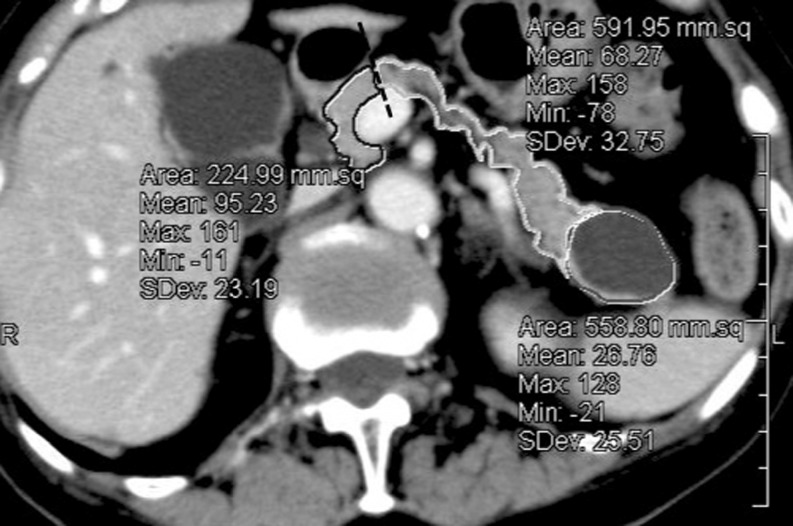
MDCT pancreas volumetry. Outlined areas are the remnant parenchyma (*black outline*), resected parenchyma (*light gray outline*), and tumor (*dark grey outline*), excluding vessels. The *dashed line* is the pancreatic resection line. To determine percent resected volume, the volume (in milliliters) of the pancreatic parenchyma per slice was calculated as the product of the pancreas area (in square millimeters) times the slice thickness (in millimeters)

Excluding tumors, cystic lesions, any dilation in the pancreatic duct and bile duct, and vessels, we outlined the borders of the pancreatic parenchyma and the resection lines on every CT slice, and we then computed the resected and remnant areas of pancreatic parenchyma for each slice (Fig. 1). The volume (in milliliters) of the pancreatic parenchyma per slice was calculated as the product of the pancreas area (in square millimeters) times the slice thickness (in millimeters). Resected and remnant volumes of the pancreatic parenchyma were computed as the sum of the slice volumes. PRV was determined using the following formula: ${\mathrm{PRV}}\left( \% \right) = \left[ {{\mathrm{resected}}\,{\mathrm{volume}}\,{\mathrm{of}}\,{\mathrm{normal}}\,{\mathrm{pancreas}} \div {\mathrm{total}}\,{\mathrm{volume}}\,{\mathrm{of}}\,{\mathrm{normal}}\,{\mathrm{pancreas}}} \right] \times 100$. PRV was calculated in 52 cases. In the remaining nine cases, this value could not be measured accurately because of pancreatic edema or tumors with unclear borders that had invaded peri-pancreatic organs.

### Definition of Postoperative New-Onset Diabetes

Postoperative new-onset diabetes was diagnosed retrospectively based on the WHO criteria of FPG ≥ 126 mg/dl detected on two or more separate days, or this abnormal FPG level detected once and plasma glucose ≥ 200 mg/dl measured in 2 h after a 75-g glucose drink. The onset day of diabetes was defined as the latter day on which abnormal blood test results were detected. In this study population, no patient was administered anti-diabetic therapy with oral agents or insulin before the development of diabetes, as defined by the criteria of this study.

### Statistical Analysis

Patient characteristics are reported as means ± standard deviation (SD), and results are presented as means ± standard error (SE) or, where indicated, medians (range). Categorical variables are expressed numerically as percentages. For analyses of repeated measurements of body weight, serum albumin, FPG, and serum HbA1c prior to and 3, 6, and 12 months after surgery, we used an analysis of variance (ANOVA) and the Mauchly test, which evaluates the sphericity assumption. We used the Student’s *t* test or Mann–Whitney test for continuous variables and Fisher’s exact test for categorical variables. A multiple logistic regression analysis yielding odds ratios and 95 % confidence intervals (CIs) was used to identify risk factors for postoperative new-onset diabetes (with *p* < 0.05). The optimal HbA1c and PRV cutoffs for predicting the occurrence of postoperative new-onset diabetes were estimated using receiver operating characteristic (ROC) curves. All analyses were performed using JMP 9.0 for Macintosh (SAS Institute Inc, Cary, NC, USA).

## Results

### Patients’ Characteristics

Physiological characteristics of the study patients are outlined in Table 1. While no patient met the WHO criteria for diabetes preoperatively, nine had impaired fasting glucose (IFG), defined as FPG of 110–125 mg/dl. The indications for DP included pancreatic tumors in 55 of the 61 patients (90 %, 25 malignant and 30 benign tumors), alcohol-induced chronic pancreatitis in three patients, autoimmune pancreatitis mimicking pancreatic cancer in two patients, and a pseudocyst following acute pancreatitis in one patient. Three patients had a first-degree family history of type 2 diabetes.

**Table 1:** Clinical characteristics of 61 non-diabetic patients who underwent distal pancreatectomy

Male patients	24 (39)
Age (years)	62 ± 14
BMI (kg/m^2^)	21.2 ± 3.8
Preoperative HbA1c (%)	5.8 ± 0.41
Preoperative IFG	9 (16)
Preoperative albumin (mg/dl)	4.0 ± 0.64
Preoperative total cholesterol (mg/dl)	182 ± 45
Preoperative pancreatic alpha-amylase (IU/l)	59 (4–264)
Operative time (min)	333 ± 88
Intraoperative blood loss (ml)	427 (5–3524)
Malignancy	25 (41)
Percent resected volume (%)	38 ± 17
POPF ≥ grade B	16 (26)
Postoperative complication ≥ Clavien’s grade II	22 (36)
Postoperative hospital stay (days)	18 (7–58)
Mortality (%)	0

Values are means ± SD, medians (range), or *n* (%)

*HbA1c* hemoglobin A1c, *IFG* impaired fasting glucose, *FPG* fasting plasma glucose, *POPF* postoperative pancreatic fistula

### Pancreas Volumetry

MDCT imaging volumetric data showed a wide range of volumes of whole, remnant, and resected pancreatic parenchyma and of tumors in patients with or without new-onset diabetes (Table 2). While the mean PRV for all 61 cases was 38 % (range 9–85 %), the mean PRV for the new-onset diabetes group was 49 %, which was significantly higher than the PRV of 32 % for the non-diabetic group.

**Table 2:** CT volumetry in DP patients

	All patients	New-onset diabetes group	No new-onset diabetes group	*p* value
Number of patients with PRV data	52	20	32	
Whole normal parenchyma (ml)	56.6 (16.0–128.2)	54.6 (27.0–89.4)	56.6 (16.0–128.2)	0.58
Remnant normal parenchyma (ml)	36.5 (4.4–116.4)	25.1 (4.4–65.8)	38.6 (6.6–116.4)	0.047
Resected normal parenchyma (ml)	18.7 (3.5–57.7)	25.8 (9.9–57.7)	16.5 (3.5–55.3)	0.004
Tumor or cystic lesion (ml)	5.4 (0–543.7)	4.1 (0–38.7)	7.4 (0.2–543.7)	0.11
PRV (%)	38 ± 17 (9–85)	49 ± 15 (20–85)	32 ± 15 (9–59)	< 0.001

Values are medians (range) or means ± SD (range). *p* values were obtained using Mann–Whitney *U* test, except for use of Student’s *t* test for PRV

*CT* computed tomography, *DP* distal pancreatectomy

### Sequential Changes in Diabetic and Nutritional Status After Surgery

We compared four physiological parameters in new-onset diabetic versus non-diabetic patients at four time points: before and 3, 6, and 12 months after surgery. Three months after surgery, there were significant increases in FPG and HbA1c in both groups (Table 3). Disturbances in glucose control occurred within the first 3 months after surgery, and did not significantly progress after that time in either group.

**Table 3:** FPG and serum HbA1c before and 3, 6, and 12 months after surgery

	All patients (*n* = 61)	*p* value	New-onset diabetes group (*n* = 22)	*p* value	No new-onset diabetes group (*n* = 39)	*p* value
FPG (mg/dl)						
Before surgery	96 ± 1.9		101 ± 3.8		93 ± 1.9	
3 months after surgery	109 ± 2.8	<0.001^a^	121 ± 5.3	0.008^a^	102 ± 2.6	0.006^a^
6 months after surgery	111 ± 4.9	0.29^b^	133 ± 11.6	0.17^b^	99 ± 2.4	0.75^b^
12 months after surgery	114 ± 5.8	0.19^b^	137 ± 12.2	0.15^b^	100 ± 3.0	0.55^b^
HbA1c (%)						
Before surgery	5.8 ± 0.05		6.1 ± 0.06		5.6 ± 0.06	
3 months after surgery	6.2 ± 0.10	<0.001^a^	6.7 ± 0.19	0.003^a^	5.9 ± 0.07	0.002^a^
6 months after surgery	6.3 ± 0.24	0.73^b^	7.0 ± 0.51	0.81^b^	5.9 ± 0.07	1.00^b^
12 months after surgery	6.4 ± 0.18	0.13^b^	7.1 ± 0.36	0.49^b^	5.9 ± 0.06	0.059^b^

Values are means ± SE. Data were analyzed using Student’s paired *t* test for each group

^a^Differences compared to values before surgery

^b^Differences compared to values at 3 months after surgery

During the post-DP follow-up period (median 26 months, range 3–88 months), 22 patients (36 %) developed new-onset diabetes (median onset time 8 months, range 0.5–42 months). In most of the 39 patients without new-onset diabetes, FPG and HbA1c increased significantly during the follow-up period; however, values remained stable in eight of these 39 patients (change in HbA1c ≤ 0.1 %), and one patient displayed improvement in glycemic control, as exhibited by a 0.4 % decrease in HbA1c.

**Fig. 2:**
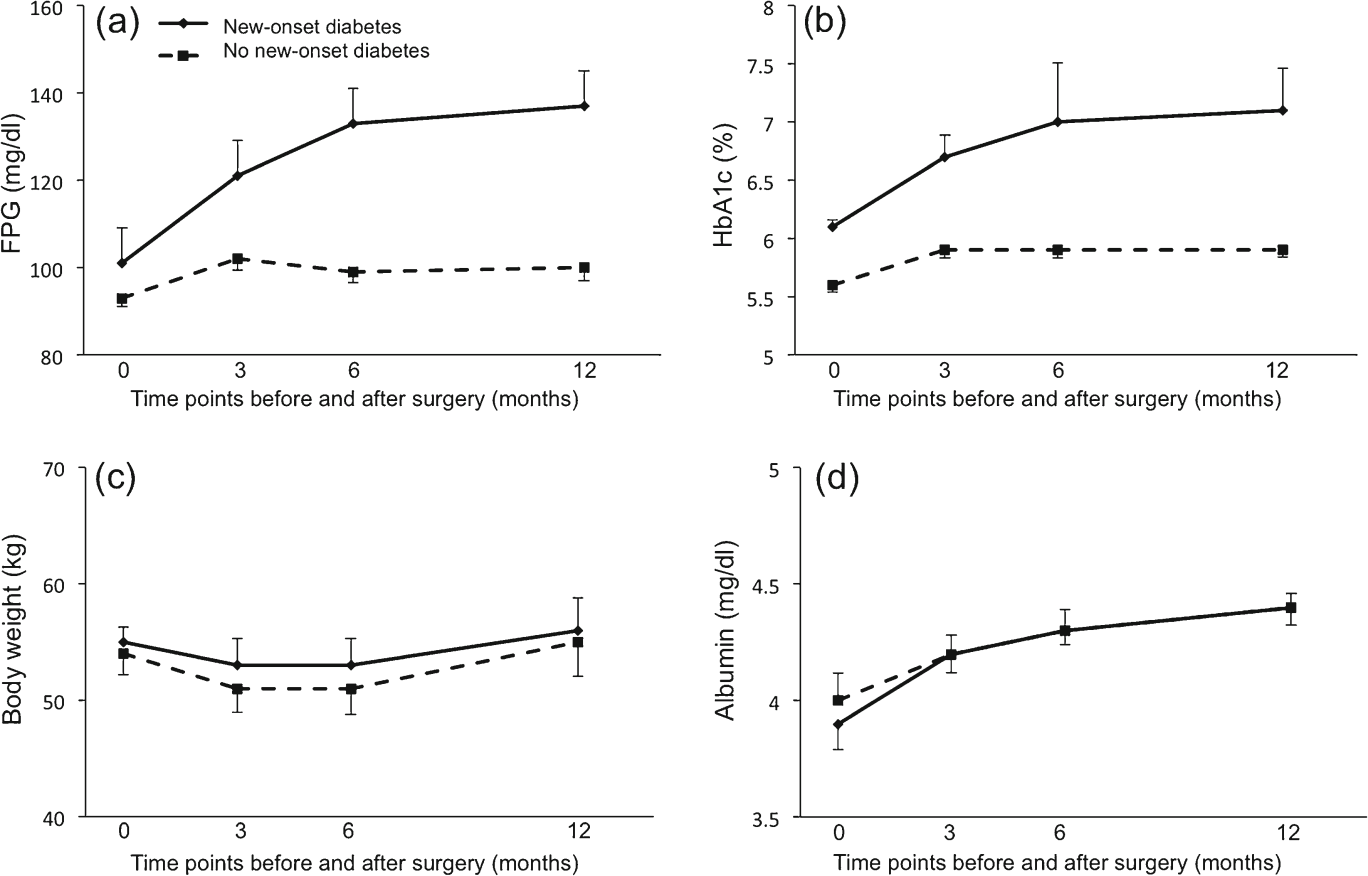
Changes in parameters before and 3, 6, and 12 months after surgery: **a** FPG, **b** HbA1c, **c** body weight, and **d** serum albumin. Values from patients who developed new-onset diabetes (*solid lines*) were compared with non-diabetic patients (*dashed lines*) using ANOVA, with evaluation of the sphericity assumption by the Mauchly test. Postoperatively, there were significant between-group differences in changes in FPG (*p* = 0.003) and HbA1c levels (*p* < 0.001). No significant between-group difference in body weight *(p* = 0.36) or albumin (*p* = 0.58) was observed

While we observed significant between-group differences in the changes in FPG (Fig. 2a) (*p* = 0.003) and HbA1c (Fig. 2b) (*p* < 0.001) over time, there were no significant differences in changes in body weight (Fig. 2c) (*p* = 0.36) or serum albumin (Fig. 2d) (*p* = 0.58). Because Mauchly tests for the sphericity assumption were not significant for these factors (*P* = 0.21, 0.82, 0.28, and 0.30, respectively), the reported *p* values are for univariate ANOVA.

### Risk Factors for Postoperative New-Onset Diabetes

Univariate analyses identified three statistically significant risk factors for postoperative new-onset diabetes: preoperative HbA1c ≥ 5.7 %, PRV > 44 %, and age (Table 4). Multivariate logistic regression analysis also identified HbA1c ≥ 5.7 % [odds ratio 15.6 (95 % CI 2.80–147), *p* = 0.001] and PRV > 44 % [odds ratio 11.3 (95% CI 2.12–92.1), *p* = 0.004] as independent risk factors for postoperative new-onset diabetes (Table 5). Regarding family history, one of three patients with first-degree family history of type 2 diabetes, of whom PRV was 85 %, developed new-onset diabetes at 2 months after surgery. We assessed the sensitivity and specificity of the HbA1c and PRV parameters using the ROC curves. The areas under the ROC curves were 0.831 for HbA1c and 0.793 for PRV. Using these curves, HbA1c of 5.7 % and PRV of 44 % were determined to be the cutoffs for predicting the occurrence of postoperative diabetes. The sensitivity, specificity, and positive and negative predictive values derived from these curves were 0.82, 0.64, 0.56, and 0.86 for HbA1c and 0.75, 0.81, 0.71, and 0.84 for PRV, respectively.

**Table 4:** Univariate analysis of risk factors for postoperative new-onset diabetes

	New-onset diabetes group (*n* = 22)	No new-onset diabetes group (*n* = 39)	*p* value
Male patients	9 (41)	15 (38)	1.00^a^
Age (years)	67 ± 3.0	59 ± 2.2	0.025^b^
BMI (kg/m^2^)	22 ± 0.78	20 ± 0.59	0.075^b^
Preoperative HbA1c ≥ 5.7 %	18 (82)	14 (36)	0.001^a^
Preoperative IFG	6 (27)	3 (8)	0.059^a^
Preoperative albumin (mg/dl)	3.9 ± 0.13	4.0 ± 0.10	0.59^b^
Preoperative total cholesterol (mg/dl)	176 ± 9.6	186 ± 7.3	0.42^b^
Preoperative pancreatic alpha-amylase (IU/l)	58 (6–243)	59 (4–264)	0.98^c^
Operative time (min)	312 ± 19	347 ± 15	0.14^b^
Intraoperative blood loss (ml)	372 (5–1125)	527 (5–3524)	0.17^c^
Malignancy	8 (36)	17 (44)	0.79^a^
PRV > 44 %	15 (75)	6 (19)	<0.001^a^
POPF ≥ grade B	6 (27)	10 (26)	1.00^a^
Postoperative complication ≥ Clavien’s grade II	7 (32)	15 (38)	0.78^a^
Postoperative hospital stay (days)	16 (7–54)	18 (7–58)	0.60^c^
Adjuvant chemotherapy	7 (32)	15 (38)	0.78^a^

Values are means ± SE, medians (range) or *n* (%)

*HbA1c* hemoglobin A1c, *FPG* fasting plasma glucose, *POPF* postoperative pancreatic fistula

^a^ *p* values were obtained using Fisher’s exact test

^b^ *p* values were obtained using Student’s *t* test

^c^ *p* values were obtained using Mann–Whitney *U* test

**Table 5:** Multivariate logistic regression analysis of risk factors for postoperative new-onset diabetes

	Odds ratio	95 % CI	*p* value
Age	1.03^a^	0.96–1.11	0.42
Preoperative IFG	1.52	0.18–14.7	0.69
Preoperative HbA1c ≥ 5.7 %	15.6	2.80–147	0.001
PRV > 44 %	11.3	2.12–92.1	0.004

^a^Odds ratio by 1 year post-DP

## Discussion

We report here two major findings from this study of patients who underwent DP. First, in the majority of preoperatively non-diabetic patients, DP led to disturbances in glucose metabolism, and there was a 36 % incidence of new-onset diabetes postoperatively. Second, in DP patients, PRV and preoperative HbA1c were independent risk factors for new-onset diabetes.

Our results enable us to provide evidence-based preoperative counseling and individualized postoperative surveillance. Prior to surgery, we can now offer patients specific information about their individual risk of postoperative diabetes. Postoperatively, appropriate surveillance may detect the development of impaired glucose metabolism [i.e., impaired glucose tolerance (IGT), IFG, and diabetes] at an early stage and enable early intervention. Intensive glucose control has been reported to decrease the risks of major cardiovascular events and death in patients with newly diagnosed type 2 diabetes.^18^ Also, in patients with IGT who are pre-diabetic, the early introduction of anti-diabetic agents has been reported to diminish the development of type 2 diabetes.^19^ The American Diabetes Association recommends intensive annual monitoring, lifestyle modification, and sometimes use of anti-diabetic agents in patients with IGT, IFG, or HbA1c of 5.7–6.4 % for the prevention and delay of developing type 2 diabetes.^20^ Therefore, early detection and intervention for endocrine insufficiency are essential for DP patients.

Prior reports have estimated the incidence of new-onset diabetes after DP at between 9 and 38 % of preoperatively non-diabetic patients.^4^,^6^,^8^,^21^ The numerous limitations of these studies (such as unspecified preoperative diabetic status of study patients, inconsistent standards for diagnosis of postoperative diabetes, and selection of cohorts of patients with chronic pancreatitis) make it difficult to evaluate the basis for this wide range of diabetes incidence. In the current study, the incidence of postoperative new-onset diabetes in preoperatively non-diabetic patients was 36 %. We attribute this relatively high measure of incidence to our application of a definitive classification system and close follow-up.

The results of this study identify PRV > 44 % as an independent risk factor for postoperative new-onset diabetes in preoperatively non-diabetic DP patients. Although beta cell mass has previously been reported to be significantly related to plasma glucose control,^14^,^22^,^23^ volumetric assessments in relation to postoperative endocrine function of the pancreas remain scarce. Previous studies in large animals^13^,^15^ and humans^22^ have demonstrated that a 50 % loss in beta cells elevates plasma glucose. The DP procedure is often referred to as a “hemi-pancreatectomy,” with an estimated 50 % reduction in pancreatic volume after transection on the superior mesenteric vein (SMV).^9^,^11^ In our study, the median PRV in 29 patients with transection on the SMV was 46 %, but we observed a wide range of values in these cases (PRV from 18 to 67 %), as well as among all cases of DP (PRV from 9 to 85 %). Variations in PRV can also be attributed to differences in the patients’ pancreatic sizes and shapes, as well as differences in pancreatic tumor characteristics (i.e., location and the involvement of the main pancreatic duct that causes atrophy of the distal pancreas). Our use of MDCT-based measurements of pancreas volume resulted in more precise PRV values and thereby provides evidence that greater resection of pancreatic tissue increases the incidence of new-onset diabetes in preoperatively non-diabetic DP patients. Thus, although pancreatic resection must be tailored to suit the tumor character (benign or malignant), location, and extent of tumor invasion, our data suggest that parenchyma-sparing pancreatectomies (such as middle pancreatectomy or tumor enucleation) are more likely to maintain postoperative pancreatic endocrine function and reduce the risk of diabetes.

In this study, we frequently observed a delay in diabetes onset in the 22 new-onset diabetics (median time 8 months, range 0.5–42 months), with only five showing signs of diabetes within 3 months. However, both groups of DP patients displayed significant increases in FPG and HbA1c levels within 3 months following surgery, but without further increases thereafter. These data lead us to hypothesize that, while surgical reduction of pancreatic parenchyma volume quickly impairs glucose metabolism, the observed lag in diabetes onset depends on other factors, such as the amount and overall health of the remaining endocrine pancreas that control plasma glucose.

In patients with insufficient functional reserve of the remnant pancreas to compensate for beta cell deficit (with severity depending on the volume of the pancreas removed), overt diabetes would develop in the early postoperative period (within 3 months postoperatively). The functional reserve of the endocrine pancreas could be estimated based on the preoperative HbA1c value, which was identified as a risk factor predictive of postoperative diabetes in this study. In patients with late-onset diabetes (later than 3 months after surgery), beta cell compensation would considerably influence the diabetes onset time. In the field of islet cell transplants, although obese individuals are generally at high risk of diabetes, it has been reported that the high demand for insulin in obese donors without diabetes promotes the necessary increase in islet cell hypertrophy and proliferation.^24^ Islet cells that remain after pancreatectomy are likely in a similar situation that stimulates islet hypertrophy and thus a compensatory increase in insulin secretion in the endocrine pancreas. Eventually, however, this pre-diabetic state may progress to overt diabetes once the endocrine pancreas is exhausted and fails to control glucose homeostasis. Also, additional factors that vary the timing of late-onset postoperative diabetes include normal progression of underlying diseases, such as pancreatitis, as well as acquired risk factors for type 2 diabetes (e.g., weight gain or aging).

Our analyses identified preoperative HbA1c as a second risk factor for postoperative new-onset diabetes. HbA1c is increasingly viewed as a superior index of chronic hyperglycemia relative to plasma glucose (which varies during the day), and the American Diabetes Association recently added HbA1c ≥ 6.5 % to its diagnostic criteria for the detection of early diabetes, with slightly lower HbA1c values (from 5.7 to 6.4 %) categorized as signaling an increased risk of diabetes.^1^ In our study, the cutoff value at which HbA1c became a risk factor was 5.7 %. Among the study’s 61 patients (none of whom met the WHO criteria for diabetes, FPG ≥ 126 mg/dl), preoperative HbA1c was ≥6.5 % in two patients and between 5.7 and 6.4 % in another 30 patients. Of these 32 individuals, 18 patients (56 %) developed post-operative diabetes. However, it is noteworthy that four additional patients with HbA1c < 5.7 %, but relatively high PRV (from 47 to 61 %), also developed diabetes.

The metabolic consequences of pancreatic resection are multifaceted and can be affected by glucoregulatory hormone concentrations, the balance between production and utilization of glucose, changes in insulin sensitivity and nutritional status, surgical complications,^25^,^26^ and tumor character (malignant or benign). Despite decreases in insulin secretion, some studies have reported post-pancreatectomy improvements in glucose control in malignant cases.^27^,^28^ Because malignant pancreatic tumors are known to produce substances that impair the action of insulin and decrease insulin sensitivity, a patient’s diabetic status can sometimes improve after tumor removal.^29^,^30^ Of the nine patients in our study who showed stable glycemic control (eight with HbA1c ≤ 0.1 %) or improved control (one with HbA1c reduced by 0.4 %) during the post-DP follow-up period, five had malignant tumors. The one patient with improved glycemic control had a highly advanced cancer, as well as preoperative HbA1c and BMI levels of 6.2 % and 28.7 kg/mm^2^, respectively, indicating pre-diabetes. This patient lost 13 % of his body weight within 3 months after surgery, and it is likely that this loss helped suppress the progression of type 2 diabetes. Our results did not demonstrate any influence of malignant tumors on postoperative endocrine function.

## Conclusion

Adequate preoperative functional reserve of the endocrine pancreas (HbA1c < 5.7 %) and maximizing the volume of the pancreatic parenchyma preserved are two key determinants of successful postoperative glycemic control. Our findings enable reliable preoperative evaluation of the risk of developing diabetes and to perform postoperative surveillance appropriately. Late-onset diabetes needs to be recognized as a common sequela of DP, and longitudinal follow-up and preventive intervention (weight control and anti-diabetic agents for pre-diabetic patients) should be introduced in high-risk patients.
